# Genome-Wide Screening and Identification of New *Trypanosoma cruzi* Antigens with Potential Application for Chronic Chagas Disease Diagnosis

**DOI:** 10.1371/journal.pone.0106304

**Published:** 2014-09-16

**Authors:** João Luís Reis-Cunha, Tiago Antônio de Oliveira Mendes, Rodrigo de Almeida Lourdes, Daihana Rodrigues dos Santos Ribeiro, Ricardo Andrez Machado-de-Avila, Maykon de Oliveira Tavares, Denise Silveira Lemos, Antônia Cláudia Jácome Câmara, Carlos Chavez Olórtegui, Marta de Lana, Lúcia Maria da Cunha Galvão, Ricardo Toshio Fujiwara, Daniella Castanheira Bartholomeu

**Affiliations:** 1 Departamento de Parasitologia, Instituto de Ciências Biológicas, Universidade Federal de Minas Gerais, Belo Horizonte, Brazil; 2 Departamento de Bioquímica e Imunologia, Instituto de Ciências Biológicas, Universidade Federal de Minas Gerais, Belo Horizonte, Brazil; 3 Departamento de Análises Clínicas, Escola de Farmácia, Universidade Federal de Ouro Preto, Ouro Preto, Brazil; 4 Programa de Pós-Graduação em Ciências da Saúde, Universidade Federal do Rio Grande do Norte, Natal, Brazil; Tulane University, United States of America

## Abstract

The protozoan *Trypanosoma cruzi* is the etiologic agent of Chagas disease, an infection that afflicts approximately 8 million people in Latin America. Diagnosis of chronic Chagas disease is currently based on serological tests because this condition is usually characterized by high anti-*T. cruzi* IgG titers and low parasitemia. The antigens used in these assays may have low specificity due to cross reactivity with antigens from related parasite infections, such as leishmaniasis, and low sensitivity caused by the high polymorphism among *T. cruzi* strains. Therefore, the identification of new *T. cruzi*-specific antigens that are conserved among the various parasite discrete typing units (DTUs) is still required. In the present study, we have explored the hybrid nature of the *T. cruzi* CL Brener strain using a broad genome screening approach to select new *T. cruzi* antigens that are conserved among the different parasite DTUs and that are absent in other trypanosomatid species. Peptide arrays containing the conserved antigens with the highest epitope prediction scores were synthesized, and the reactivity of the peptides were tested by immunoblot using sera from C57BL/6 mice chronically infected with *T. cruzi* strains from the TcI, TcII or TcVI DTU. The two *T. cruzi* proteins that contained the most promising peptides were expressed as recombinant proteins and tested in ELISA experiments with sera from chagasic patients with distinct clinical manifestations: those infected with *T. cruzi* from different DTUs and those with cutaneous or visceral leishmaniasis. These proteins, named rTc_11623.20 and rTc_N_10421.310, exhibited 94.83 and 89.66% sensitivity, 98.2 and 94.6% specificity, respectively, and a pool of these 2 proteins exhibited 96.55% sensitivity and 98.18% specificity. This work led to the identification of two new antigens with great potential application in the diagnosis of chronic Chagas disease.

## Introduction

The protozoan *Trypanosoma cruzi* is the etiologic agent of Chagas disease, an infection that afflicts 8 million people in Latin America, causes high morbidity and accounts for 662,000 disability-adjusted life years (DALYs) [1]–[4]. The parasite is distributed from Southern USA to Southern Argentina, comprising 22 countries, where bloodsucking insects of the Triatominae subfamily are incriminated in the vector-borne transmission route of Chagas disease [2].

Immigration and travel of chagasic patients to non-endemic countries, such as the US [5], Australia and Spain [6], has spread this infection worldwide [7], [8], highlighting other important transmission routes, such as blood transfusion, congenital transmission and organ transplantation [7], [8]. These alternative transmission routes have also gained importance in endemic countries where vector-borne transmission has been controlled [2], [9].

The progression of Chagas disease begins with an acute phase that lasts 10–90 days and is characterized by high parasitemia but is usually asymptomatic. After approximately three months of infection, the parasitemia is controlled by the host immune response, and the patients reach the chronic phase, which is usually characterized by high anti-*T. cruzi* IgG titers [10]. Chronic Chagas disease is asymptomatic in 70% of the patients but evolves into cardiac, digestive or mixed clinical forms in 30% of cases [10]–[12].

Because parasitemia is low during chronic Chagas disease, diagnosed is mainly carried out using serologic assays, such as indirect ELISA, indirect hemagglutination and indirect immunofluorescence [13]–[15]. The use of whole or fractionated parasite protein extracts as antigens has been replaced by recombinant proteins or peptides, which have higher specificity due to lower cross-reactivity with other infections, such as leishmaniasis [16]–[18] and *T. rangeli* [15], [19]. In addition, the use of recombinant proteins or peptides facilitates the standardization of methods for antigen preparation [15], [20].

The *T. cruzi* taxon has been extensively documented as highly polymorphic [21]–[24], and currently, six different DTUs (discrete typing units), named TcI – TcVI, have been recognized [25]. This high variability among *T. cruzi* strains compromises the sensitivity of serological tests for Chagas disease [15], [26]. The TcV and TcVI DTUs are derived from recent recombination events between strains from TcII and TcIII [27], [28]. CL Brener, the first strain to have its genome sequenced [29], belongs to the TcVI DTU. Because CL Brener is a hybrid strain, its genome is composed of two haplotypes named esmo-like and non-esmo-like, which are derived from TcII and TcIII, respectively [29]. TcI, TcII, TcV and TcVI are the most common lineages associated with human infections [30], [31].

In the present study, we have explored the hybrid nature of the CL Brener strain using a broad genome screening approach to select new *T. cruzi* antigens that are potentially conserved among the different parasite DTUs and that are absent in other trypanosomatid species. This analysis led to the identification of two new candidate antigens for use in the serodiagnosis of chronic Chagas disease.

## Materials and Methods

### Ethics statement

The design and methodology of all experiments involving mice were performed in accordance with the guidelines of COBEA (Brazilian College of Animal Experimentation), strictly followed the Brazilian law for “Procedures for the Scientific Use of Animals” (11.794/2008) and were approved by the animal-care ethics committee of the Federal University of Minas Gerais (protocol number 143/2009).

The study protocol involving human samples was approved by the Ethics Committee of the Federal University of Minas Gerais (UFMG) under protocol number No. 312/06. All subjects provided written informed consent before blood sample was collected.

### Mouse sera

Each experimental group was composed of five 6-8-week old C57BL/6 female mice, which were infected intraperitoneally with *T. cruzi* bloodstream trypomastigotes from Colombiana, Y or CL Brener strains. All parasites were previously genotyped according to Souto *et al*., 1996 [32], de-Freitas *et al*., 2006 [28] and Burgos *et al*., 2007 [33]. Infection was confirmed by the observation of trypomastigote forms in blood collected from the infected mice's tail seven days after the parasite inoculation. An additional group was infected with *T. rangeli* trypomastigotes from the SC-58 strain, and infection was confirmed by PCR [34]. Six uninfected mice were used as the control group. The chronic phase of infection was confirmed 4 months post-infection by negative parasitemia and the presence of anti-parasite IgG (as tested against *T. cruzi* and *T. rangeli* crude antigens) by ELISA [35]. Blood from chronically infected mice was then collected by cardiac puncture followed by 30 minutes incubation at room temperature and centrifugation at 4,000 x *g* for 15 minutes at 4°C to obtain serum, which was stored at −80°C until use.

We performed ELISA using sera from mice chronically infected with *T. cruzi* and the epimastigote raw extract as antigen and determined that the 1∶100 dilution would be adequate to discriminate between sera from infected and non-infected mice.

### Human sera

A total of 58 sera samples from chagasic patients were used in this study. Of these samples, 43 were from chagasic patients infected with untyped parasites collected from Rio Grande do Norte and Minas Gerais States, Brazil, 8 were samples from chagasic patients previously characterized to be infected with TcII [36], and 7 were samples from patients known to be infected with TcVI. Of the 43 samples from chagasic patients infected with untyped parasites, 23 are from patients with defined clinical forms of Chagas disease: 9 are from patients with indeterminate forms, 14 with chronic chagasic cardiopathy [37]. Infection was confirmed by testing the reactivity of sera from chagasic patients using Chagatest recombinant ELISA v. 3.0 kit, Chagatest hemagglutination inhibition (HAI), screening A-V kit (Wiener Lab, Rosário, Argentina) using titers of 1∶40 as the cutoff value, and indirect immunofluorescence (IIF) using a *T. cruzi* Y strain epimastigotes maintained in culture and fixed with 20% formaldehyde as the antigen all according to the manufacturer's instructions. Anti-human IgG immunoglobulin labeled with fluorescein isothiocyanate (Sigma Chemical Company, Missouri, USA) was used as the secondary antibody at titers of 1∶40 as the cutoff value [38]. The serum samples were considered positive for *T. cruzi* infection when two methods with different principles were reactive, indeterminate when only one method was reactive, and negative when these methods were nonreactive in accordance with the recommendations of the World Health Organization [39]. Sera exhibiting indeterminate results were evaluated using western blot (TESAcruzi, bioMérieux Brazil) to measure anti-*T. cruzi* reactivity [40].

To evaluate the cross-reactivity with leishmaniasis, sera from 5 patients with clinical signs of cutaneous leishmaniasis and confirmed infections by microscopic analysis of biopsy from the cutaneous lesion from the Centro de Referência em Leishmaniose in Januária/MG Brazil were used. In addition, sera from 5 patients with visceral leishmaniasis with reactivity confirmed by serology, molecular analysis and microscopic analysis of bone marrow aspirates from Hospital Universitário Clemente de Faria in Montes Claros/MG Brazil were used.

The sera of 45 healthy Brazilian individuals residing in Belo Horizonte, Minas Gerais were used as a negative control.

### *In silico* prediction of linear B-cell epitopes

Linear B-cell epitope predictions were performed on all predicted proteins in CL Brener genome release 4.1 [29] using the BepiPred 1.0 program with a cutoff of 1.3 [41] as previously described [42]. To select epitopes that are potentially conserved among *T. cruzi* strains, the predicted proteome from the esmo-like and non-esmo-like haplotypes of the *T. cruzi* CL Brener strain [29] were aligned using the ClustalW program [43]. The predicted epitopes that had identical sequences in the two CL Brener haplotypes were selected, and 15–18 mer peptides with mean score ≥1.3 were retained. To reduce the chance of cross-reaction with other parasites, the selected peptides were analyzed with BLASTP searches against the predicted *L. braziliensis*, *L. infantum* and *L. major* proteomes, and those that showed greater than 70% similarity along 70% of the length were discarded. After analysis, 450 peptides with the highest mean BepiPred score that were identical between the two CL Brener haplotypes and absent in the predicted *Leishmania major*, *L. braziliensis* and *L. infantum* proteomes were selected for synthesis.

### Spot synthesis and immunoblotting

Peptide arrays containing 30, 120 or 300 distinct peptides (450 in total) were synthesized as previously described [42] (Table S1). These membranes were used in immunoblotting assays with pools of sera from five C57BL/6 mice chronically infected with Colombiana (TcI), Y (TcII) or CL Brener (TcVI) *T. cruzi* strains or uninfected mice. Briefly, the membranes were blocked for 16 hours with 5% BSA, 4% sucrose in PBS, washed 3 times for 10 minutes with 0.1% Tween-20 in PBS and incubated with one of the pools of sera diluted 1∶2,500 in 0.1% Tween in PBS for one hour. Subsequently, the membranes were washed as described above, incubated with horseradish peroxidase-conjugated anti-mouse IgG (Sigma-Aldrich) diluted 1∶35,000 in 0.1% Tween in PBS for one hour, washed as described above and visualized by chemiluminescence using ECL Plus western blotting detection system (GE-Healthcare) with 20 minutes exposure on an ImageQuant LAS 4000 digital imaging system (GE-Healthcare). After data acquisition, the membranes were regenerated for use with another pool of sera. The regeneration was performed by washing the membranes 3 times for 10 minutes with dimethylformamide, followed by incubation for 16 hours with an 8 M urea, 10% SDS solution. The membranes were then washed twice for 30 minutes in the 8 M urea, 10% SDS solution, washed once with deionized water, and then washed 3 times in 55% ethanol and 10% acetic acid. Lastly, the membranes were washed for 5 minutes with deionized water. The densitometric value of each spot was calculated using ImageMaster 2D Platinum software (GE-Healthcare). The relative value (RV) for each spot was calculated using the formula RV = PDV/NDV, where PDV corresponds to the spot densitometric value when a positive pool of sera were used and NDV corresponds to the densitometric value when the negative pool of sera were used. Spots with RV value of 2 or higher were considered reactive.

### Expression and purification of recombinant proteins

The primer sequences used to amplify the entire coding sequence of the Tc00.1047053511623.20 gene and the 5′ end of the Tc00.1047053510421.310 gene are listed in Table S2. The PCR reactions were performed using 100 ng of genomic DNA from the *T. cruzi* CL Brener strain and Platinum Taq DNA High Fidelity Polymerase (Invitrogen) according to the manufacturer's instructions. The amplicons were purified from an agarose gel using the Illustra GFX PCR DNA and Gel Band Purification Kit (GE Healthcare) according to the manufacturer's instructions. The purified fragments were cloned into the pET-28a-TEV expression vector (CeBiMe, Campinas/SP) in frame with the N-terminal poly-histidine tag. The constructs were sequenced to confirm that the fragments were cloned in frame, and the recombinant proteins were expressed in the *Escherichia coli* BL-21Star strain by adding 1 mM isopropyl-β-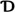-thiogalactopyranoside (IPTG) to the culture for 3 h at 37°C shaking at 180 rpm. Recombinant proteins were purified using affinity chromatography with HisTrap HP 5 mL columns (GE Healthcare Life Sciences) in the ÄKTA primeplus (GE Healthcare Life Sciences) system. The purified proteins were separated by SDS-PAGE and visualized by western blotting to confirm their identity and purity (data not shown).

### ELISA

ELISA half area plates (Greiner-Bio-One-675061) were coated with 50 µL/well of a solution containing 5 µg/mL recombinant Tc00.1047053511623.20 (*r*Tc_11623.20) protein, 10 µg/mL recombinant protein corresponding to the N-terminal portion of the Tc00.1047053510421.310 protein (*r*Tc_N_10421.310), or 10 µg/mL protein mixture containing both recombinant proteins at a ratio of 1∶1 diluted in ultrapure H_2_O for 18 h at 4°C. Plates were blocked with 200 µL 5% BSA in PBS for 1 hour at 37°C. The human or mouse sera, diluted 1∶100 in 2.5% BSA in PBS was added and incubated for 1 hour at 37°C. The plates were washed four times with 200 µL PBS containing 0.05% Tween 20 and incubated for 1 hour at 37°C with 50 µL secondary horseradish peroxidase-conjugated anti-human or anti-mouse IgG antibody (Sigma-Aldrich) diluted 1∶5,000 in 2.5% BSA in PBS. Then, the plates were washed four times as previously described and incubated for 15 minutes at 37°C in a dark room with 50 µL revealing solution (0.1 M citric acid, 0.2 M Na_2_PO_4_, 0.05% OPD, 0.1% H_2_O_2_). The reaction was interrupted by adding 50 µL 4N HCl, and the absorbance was measured at 490 nm in an automated Versa Max Microplate Reader. Each sample was assayed in triplicate.

### Statistical analysis

All statistical analyses were performed using Graph Prism 5.0 software. The normal distribution of data was evaluated by the Kolmogorov-Smirnov test, an unpaired *t*-test was used for the comparative analysis between the two data sets, and ANOVA was used to evaluate three or more experimental groups. P values lower than 0.05 were considered statistically significant. The values of sensitivity, specificity, positive predictive value (PPV), negative predictive value (NPV) and accuracy were calculated for the human sera. The cutoff value was determined based on the Receiver Operating Characteristic (ROC) curve to maximize sensibility and specificity.

## Results

### Evaluation of the reactivity of the selected peptides with the sera from C57BL/6 mice infected with different *T. cruzi* strains using immunoscreening of peptide arrays

To correctly diagnose patients with chronic Chagas disease, identifying antigens that are conserved among parasite strains but do not cross-react with sera from individuals infected with related parasites, such as *T. rangeli* and *Leishmania* species, is imperative.

As an attempt to identify epitopes conserved in distinct *T. cruzi* DTUs, we have performed polymorphism analysis and B-cell epitope prediction on all proteins derived from single copy genes represented by allele pairs in the CL Brener genome. We assumed that because CL Brener is a recent hybrid between TcII and TcIII DTUs, alleles pairs that contain conserved epitopes are likely to be conserved in the parental genotypes. To this end, we aligned 3,983 proteins derived from allele pair from esmo-like and non-esmo-like haplotypes, and the conserved sequences were analyzed by epitope prediction using the BepiPred algorithm [41]. A total of 1,488 conserved predicted B cell epitopes were identified. To reduce the chance of cross-reactivity with related parasites, we excluded 402 sequences that had high sequence similarity to the predicted proteome of human-infectious *Leishmania* species. A total of 1,086 peptides ranging from 15–18 amino acids that are potentially conserved among different *T. cruzi* strains but are absent in *Leishmania* sp. were selected.

**Figure 1:**
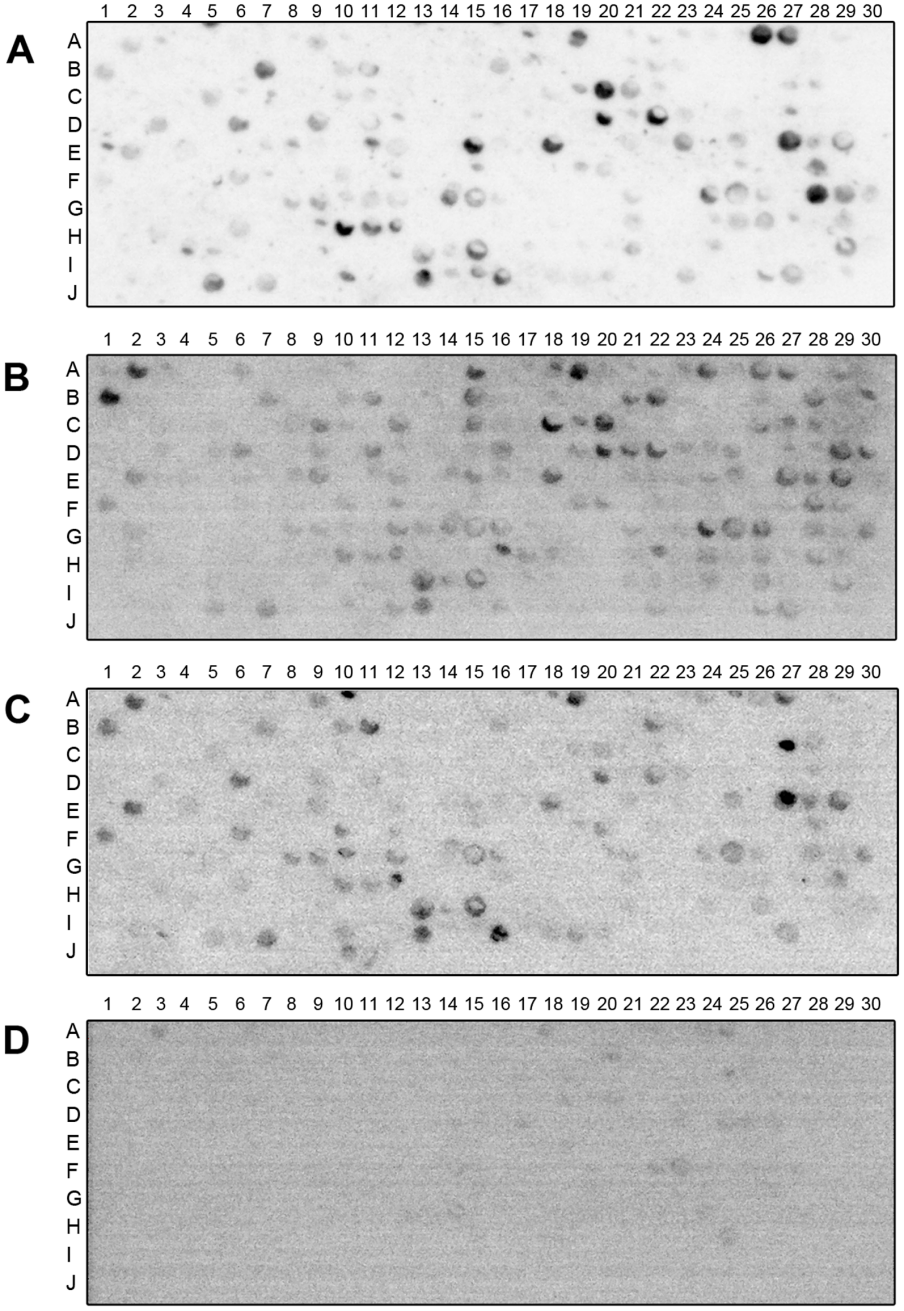
Immunoblot of a peptide array with sera from C57BL/6 mice chronically infected with different *T. cruzi* strains. (A) Sera from mice infected with Colombiana (TcI); (B), Y (TcII) or (C), CL Brener (TcVI); or (D) uninfected mice.

To determine the reactivity of the sera from mice experimentally infected with *T. cruzi* to these antigens, 450 peptides with high BepiPred scores were synthesized on cellulose membranes and probed using a pool of sera from C57BL/6 mice chronically infected with the *T. cruzi* strains Colombiana (TcI), Y (TcII) or CL Brener (TcVI) (Figure 1). These strains correspond to the *T. cruzi* DTUs commonly associated with human infections.

The antigen spots that were reactive with pooled serum from uninfected mice were excluded from further analysis. The densitometric value of each spot with each pool of serum was determined, and their relative value (RV) was calculated using the formula RV = PDV/NDV. Spots with an RV value of 2 or higher were categorized as reactive. Peptides that were reactive with at least one serum pool from *T. cruzi*-infected mice are listed in the Table S3. Two peptides, named C6-30 and E27-300, had RV higher than 2 for all pools of sera from the mice chronically infected with each one of the *T. cruzi* strains assayed. The genes that encode the proteins containing these peptides are Tc00.1047053510421.310 and Tc00.1047053511623.20, respectively.

**Figure 2:**
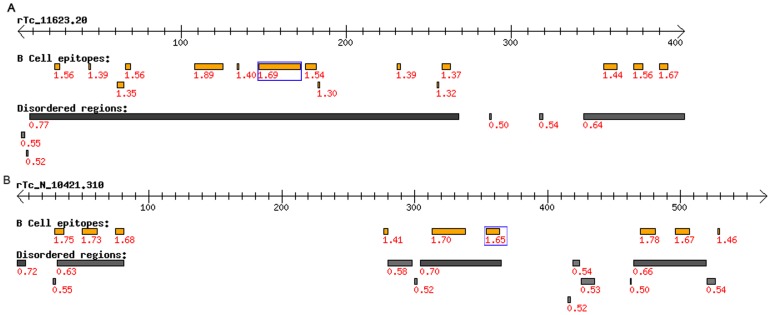
Predictions of B-cell linear epitopes and intrinsically unstructured/disordered regions in *r*Tc_11623.20 (A) and *r*Tc_N_10421.310 (B). The complete sequences of the recombinant proteins were submitted to the BepiPred and IUPred algorithms. The dashed arrow corresponds to the complete amino acid sequence of the recombinant proteins. The orange boxes indicate linear B-cell epitopes as predicted by BepiPred, and the gray boxes indicate unfolded regions that were predicted by IUPred. The epitope region that contains the peptide screened in the immunoblotting for each one of the proteins is highlighted by a blue box.

These protein sequences were analyzed for linear B cell epitopes and intrinsically unstructured regions. The presence of these unstructured regions suggest that a given protein region is in an unfolded structure and therefore possibly accessible for antibody binding. Hence, the co-occurrence of these two features reinforces the accuracy of the B cell linear epitope prediction. All selected proteins had a high density of predicted B cell epitopes and large predicted unfolded regions that co-localize with these epitopes (Figure 2).

### Expression and purification of recombinant proteins

The full-length Tc00.1047053511623.20 gene was cloned into the pET28a-TEV vector for recombinant expression. Due to large size of the Tc00.1047053510421.310 gene, only its first 1,704 nucleotides which contains the coding sequence of the peptide C6-30 were cloned into the expression vector. Both sequences were cloned in frame in the expression vector as confirmed by sequencing (data not shown). The recombinant proteins with a predicted molecular weight of 47 kDa for Tc00.1047053511623.20 (named *r*Tc_11623.20) and 66 kDa for the N-terminal portion of the protein Tc00.1047053510421.310 (*r*Tc_N_10421.310) were successfully expressed in *E. coli* BL21 cells as insoluble his-tagged proteins and purified by Ni^2+^-affinity chromatography (GE Healthcare). The recombinant protein expression was confirmed by western blot using mouse anti-His antibody (GE Healthcare) (data not shown).

### Reactivity of sera from C57BL/6 mice chronically infected with *T. cruzi* to the purified recombinant proteins

**Figure 3:**
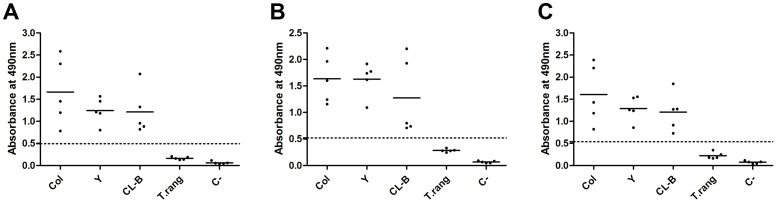
Recognition of *r*Tc_11623.20 and *r*Tc_N_10421.310 by sera from C57BL/6 mice chronically infected with different *T. cruzi* strains. Sera from mice chronically infected with CL Brener (CL-B), Colombiana (Col) or Y (Y) *T. cruzi* strains, mice infected with *T. rangeli* (T.rang) or uninfected mice (C-) were screened by ELISA with *r*Tc_11623.20 (A), *r*Tc_N_10421.310 (B) or with a pool of these two recombinant proteins (C). The dotted line represents the cutoff value that was obtained based on the ROC curve. The solid line corresponds to the mean values. Col, mice infected with Colombiana; Y, mice infected with the Y strain; CL-B, mice infected with the CL Brener; T. rang, *T. rangeli*-infected mice. C-, uninfected mice.

Initially, the recombinant proteins were analyzed by ELISA using individual sera from three groups of C57BL/6 mice: (i) mice chronically infected with Colombiana (TcI), Y (TcII) or CL Brener (TcVI) *T. cruzi* strains, (ii) mice infected with the SC-58 strain of *T. rangeli*, (iii) or uninfected mice. The serum samples used in this assay were the same as the pooled samples used for the immunoblotting screen. Both *r*Tc_11623.20 and *r*Tc_N_10421.310 had high sensitivity and specificity (Figure 3, Figure S1 and Table S4). In concordance with the immunoblotting assays, all sera from mice chronically infected with each one of the three *T. cruzi* strains were reactive with *r*Tc_11623.20 and *r*Tc_N_10421.310 when they were assayed individually and when assayed using an antigen pool containing both recombinant proteins by ELISA. The sera from *T. rangeli*-infected mice or uninfected mice exhibited results below the cutoff for all antigens (Figure 3).

### Performance of *r*Tc_11623.20 and *r*Tc_N_10421.310 for diagnosing Chagas disease by ELISA

**Figure 4:**
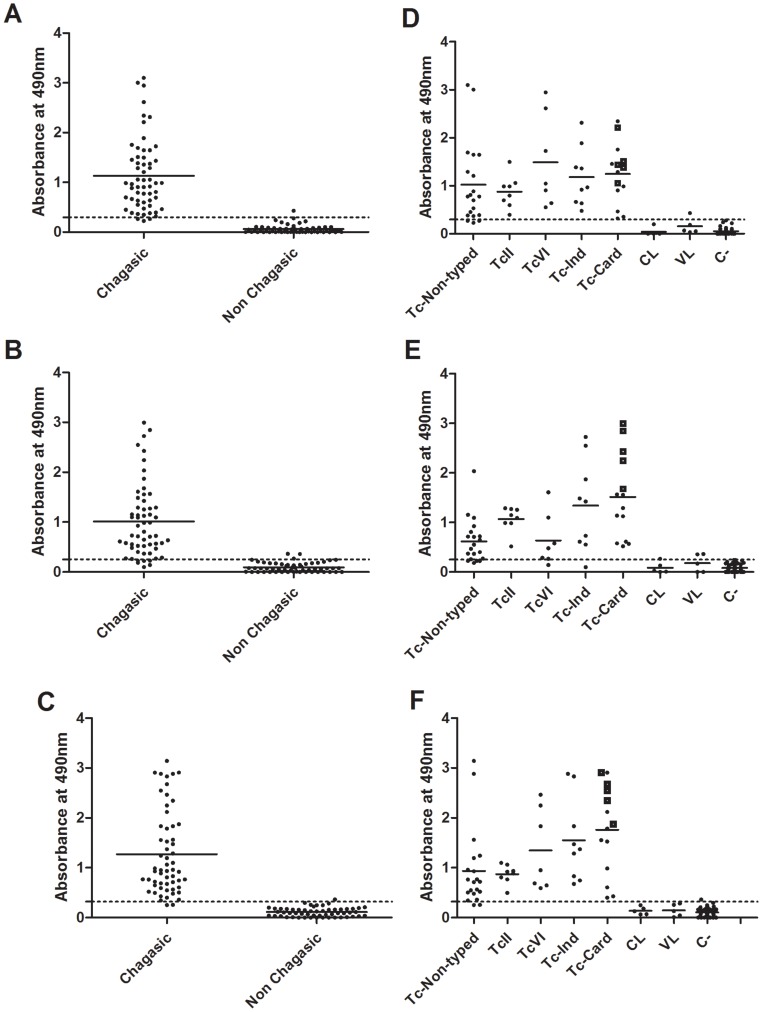
Recognition of *r*Tc_11623.20 and *r*Tc_N_10421.310 by the sera from chronic chagasic patients or *Leishmania*-infected individuals. Sera from chronic chagasic patients and sera from patients with cutaneous and visceral leishmaniasis were assayed by ELISA using *r*Tc_11623.20 (A, D), *r*Tc_N_10421.310 (B, E) and pooled recombinant proteins (C, F). Tc-non typed, sera from chronic chagasic patients; TcII, sera from patients infected with *T. cruzi* TcII DTU; TcVI, sera from patients infected with *T. cruzi* TcVI DTU, Tc-Ind, sera from chagasic patients in the indeterminate form of Chagas disease; Tc-Card, sera from chagasic patients in the cardiac stage of Chagas disease; CL, sera from patients with cutaneous leishmaniasis; VL, sera from patients with visceral leishmaniasis; C-, uninfected individuals. Squares represent sera from chagasic patient in the initial cardiac stage. The dotted line represents the cutoff value that was obtained based on the ROC curve. The solid line corresponds to the mean values.

To evaluate the accuracy of the recombinant proteins *r*Tc_11623.20 and *r*Tc_N_10421.310 in the diagnosis of chronic human Chagas disease, a total of 113 human sera, of which 58 samples correspond to chronic chagasic patients and 55 to non-chagasic control patients, were screened. All serum samples were tested by ELISA using both recombinant proteins (Figure 4, Figure S2).

Of the 58 sera from chagasic patients, 55 were reactive with *r*Tc_11623.20 (Figure 4A), 52 with *r*Tc_N_10421.310 (Figure 4B) and 56 with a pool of both recombinant proteins (Figure 4C). All three sera that were below the cutoff for *r*Tc_11623.20 belong to the non-typed group (Figure 4D). Of the six sera from chagasic patients that were below the cutoff value for *r*Tc_N_10421.310, four samples belong to the non-typed group, one to the TcVI group and one to the Tc-Card group (Figure 4E). The two sera that were below the cutoff for the pool of both recombinant proteins belong to the non-typed group (Figure 4F).

Of the 55 sera from non-chagasic individuals, 54, 52, and 54 samples were below cutoff for *r*Tc_11623.20 (Figure 4A), *r*Tc_N_10421.310 (Figure 4B) and the pool of both proteins, respectively (Figure 4C). The only non-chagasic serum sample that was above cutoff value for *r*Tc_11623.20 was from a patient with visceral leishmaniasis (Figure 4D). Of the three sera from non-chagasic individuals that had values above cutoff for *r*Tc_N_10421.310, two were from patients with visceral leishmaniasis and one from a patient with cutaneous leishmaniasis (Figure 4E). The only non-chagasic serum sample that had values above cutoff for the pooled recombinant proteins belonged to the uninfected group (Figure 4F).

The sensitivity, specificity, positive predictive value, negative predictive value and accuracy values were 94.83, 98.18, 98.30, 94.82 and 96.4, respectively for *r*Tc_11623.20; 89.66, 94.55, 95.08, 90.16, and 92.03 respectively for *r*Tc_N_10421.310 and 96.55, 98.18, 98.30, 96.49 and 97.34, respectively for the pooled antigens (Table S4).

## Discussion

The lack of highly accurate methods to diagnose Chagas disease hampers the correct identification and treatment of infected individuals and restricts the evaluation of effectiveness of any initiative aiming at blocking transmission or vaccination in endemic countries [13], [15]. Additionally, reports of blood transfusion transmission in the USA, Spain and Canada, countries that receive a high contingency of Latin-American immigrants also highlights the importance of an accurate diagnostic test to identify Chagas disease infection in samples from blood banks of non-endemic countries [5]–[8], [44], [45].

Due to the high genetic variability among the *T. cruzi* strains, the sensitivity of many diagnostic tests varies widely with sera from patients infected with parasites from different geographic regions, where different *T.cruzi* DTUs are found [31]. To identify new antigens that are potentially conserved among distinct *T. cruzi* strains, in this study, we applied a genome-wide screening aiming at identifying epitopes that are conserved between esmo-like and non-esmo haplotypes of the CL Brener hybrid strain. Because the esmo-like haplotype is derived from TcII and non-esmo haplotype is derived from the TcIII, antigens that are conserved between these two haplotypes are likely to also be conserved among other *T. cruzi* DTUs.

The major drawback of the specificity of Chagas disease serologic tests is cross-reaction with sera from patients infected with related parasites, such as *T. rangeli* and the *Leishmania* genus [15], [18], [46], [47]. In an attempt to increase the specificity of the selected epitopes, we excluded sequences that had high similarity to the predicted proteome of sequenced, human-infectious *Leishmania* species. This approach allowed us to identify two new antigens, named *r*Tc_11623.20 and *r*Tc_N_10421.310, designed for Chagas disease diagnosis.

These two antigens were first screened with the sera from C57BL/6 mice chronically infected with *T. cruzi* strains from TcI (Colombiana), TcII (Y) and TcVI (CL Brener) DTUs. The selection of these *T. cruzi* DTUs was due to their broad geographic distribution, importance in human infection in Latin America and their categorization into highly divergent *T. cruzi* DTUs [25], [31]. Both proteins, used individually or pooled, were able to discriminate 100% of the sera from mice infected with all the three *T. cruzi* strains from the uninfected mice or from mice infected with *T. rangeli* (Figure 3).

Next, we tested the accuracy of *r*Tc_11623.20 and *r*Tc_N_10421.310 as antigens to diagnose human patients with chronic Chagas disease. To this end, sera from patients infected with TcII and TcVI *T. cruzi* DTUs, sera from patients with different clinical forms of Chagas disease and sera from patients infected with non-typed *T. cruzi* were used. This sera panel was used because patients that are infected with different DTUs or present different clinical forms have been reported to exhibit discordant results when evaluated with conventional Chagas serologic tests [14], [15], [26]. Additionally, cross-reactivity between sera from patients infected with *T. cruzi* and *Leishmania* spp. is well documented [15], [17], [18]. To determine the specificity of both recombinant proteins, sera from healthy humans and patients with cutaneous or visceral leishmaniasis were also assayed.

The sensitivity and specificity of the *r*Tc_11623.20, *r*Tc_N_10421.310 and pooled recombinant proteins were comparable with the results from commercial tests as the INNO-LIA Chagas assay, which contains a combination of recombinant antigens and exhibited 99.4% sensitivity and 98.1% specificity [47]. The *r*Tc_11623.20 and *r*Tc_N_10421.310 antigens also showed slightly better results than the recombinant protein TSSA VI, which exhibits 87% sensitivity and 97.4% specificity [48]. Additionally, *r*Tc_11623.20 and *r*Tc_N_10421.310 showed similar accuracy compared to ELISA-IMT, Chagas III (BIOSChile) ELISAcruzi (bioMérieux Brasil SA), Chagatek (bioMérieux Brasil SA) Chagatest Rec v3.0 (Wiener) and Pathozyme Chagas (Omega), which have sensitivities ranging from 75 to 100% and specificities ranging from 82.84 to 100% [15].

It is well established that new potential antigens selected for the diagnosis of chronic Chagas disease must meet three criteria: (i) expression in *T. cruzi* isolates from different DTUs and absence in other infectious disease pathogens. (ii) high immunogenicity regardless the clinical form of Chagas disease and (iii) stability and adaptability to quality-control tests to guarantee reproducibility [13], [49], [50]. We believe that the antigens identified in this study meet all these criteria. First, our data suggests that they were able to discriminate sera from patients infected with *T. cruzi* from sera from patients with cutaneous, visceral leishmaniasis or healthy donors, with specificity of 98.18 for *r*Tc_11623.20, 94.55 for *r*Tc_N_10421.310 and 98.18 for a pool of both proteins. Second, the antigens were recognized by 14 out of the 15 sera from patients infected with *T. cruzi* strains belonging to different DTUs. Third, both antigens were reactive with sera from patients with different clinical forms of Chagas disease, with the exception of one indeterminate sera for the protein *r*Tc_N_10421.310. Forth, the expression protocol used in this study yielded approximately 17 mg of protein/culture liter, which allows nearly 22,500 sera trials to be performed with *r*Tc_11623.20 and 11,300 trials with *r*Tc_N_10421.310.

Further studies using a larger sera panel from negative and positive individuals with different clinical forms and from distinct endemic areas throughout Latin America where different *T. cruzi* DTUs are found will be necessary to better characterize the antigens identified in this study. Additionally, we are currently expanding the genome wide approach for the antigen selection described in this work to include new sequenced *T. cruzi* genomes [51], [52] as an attempt to define an highly effective antigenic panel for the Chagas disease serodiagnosis.

## Supporting Information

Figure S1 **ROC curves obtained from the ELISA with the recombinant antigens and the sera from C57BL/6 mice infected with** ***T. cruzi*** **,** ***T rangeli*** **or non-infected mice.**
(DOCX)

Figure S2 **ROC curves obtained from the ELISA with the recombinant antigens and sera from Chagasic and non-Chagasic humam patients.**
(DOCX)

Table S1 **Sequence of the peptides in the immunoblotting membranes.**
(XLSX)

Table S2 **Primers used to amplify the entire coding region of the Tc00.1047053511623.20 gene and the 5′ end of the Tc00.1047053510421.310 gene.**
(DOCX)

Table S3 **Reactivity of the top ten peptides in the immunoblotting assays with the sera from mice chronically infected with distinct** ***T. cruzi*** **strains.**
(DOCX)

Table S4 **Measure of diagnostic performance for rTc_11623.20, rTc_N_10421.310 and pooled antigens.**
(DOCX)
