# Supplementary material for: Genome-Wide Screening and Identification of New Trypanosoma cruzi Antigens with Potential Application for Chronic Chagas Disease Diagnosis
Source: PLoS One. 2014 Sep 16;9(9):e106304. doi: 10.1371/journal.pone.0106304 (PMC4165580; doi:10.1371/journal.pone.0106304)
Supplement: Figure S1 — ROC curves obtained from the ELISA with the recombinant antigens and the sera from C57BL/6 mice infected with T. cruzi , T rangeli or non-infected mice. (DOCX) [file pone.0106304.s001.docx]

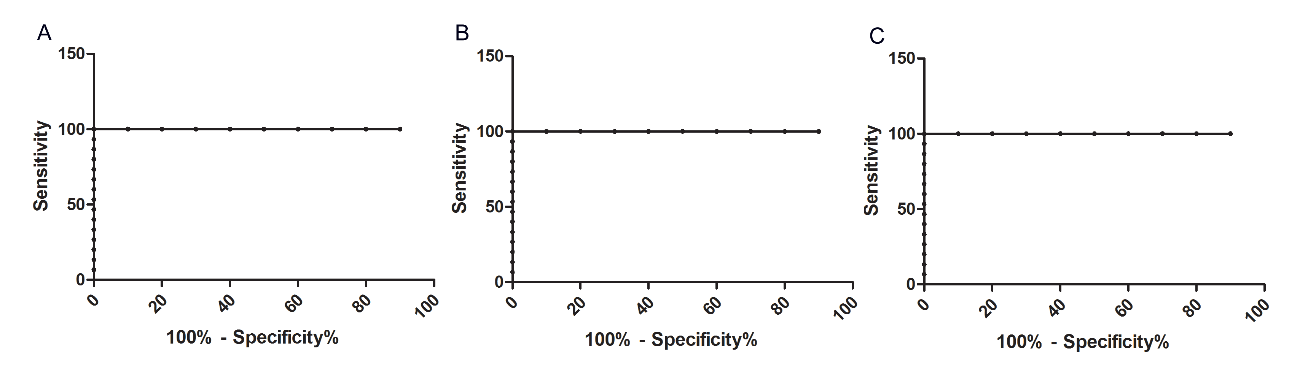


Figure S1 – ROC curves obtained from *r*Tc_11623.20 (A) and *r*Tc_N_10421.310 (B) and a pool of these two recombinant proteins (C) as antigens, with sera from C57BL/6 mice chronically infected with Colombiana (TcI), Y (TcII) and CL Brener (TcVI) *T. cruzi* strains, mice infected with *T. rangeli* or uninfected mice. These results were used to determinate the cutoff value in order to maximize sensitivity and specificity.
