# Supplementary material for: Genome-Wide Screening and Identification of New Trypanosoma cruzi Antigens with Potential Application for Chronic Chagas Disease Diagnosis
Source: PLoS One. 2014 Sep 16;9(9):e106304. doi: 10.1371/journal.pone.0106304 (PMC4165580; doi:10.1371/journal.pone.0106304)
Supplement: Figure S2 — ROC curves obtained from the ELISA with the recombinant antigens and sera from Chagasic and non-Chagasic humam patients. (DOCX) [file pone.0106304.s002.docx]

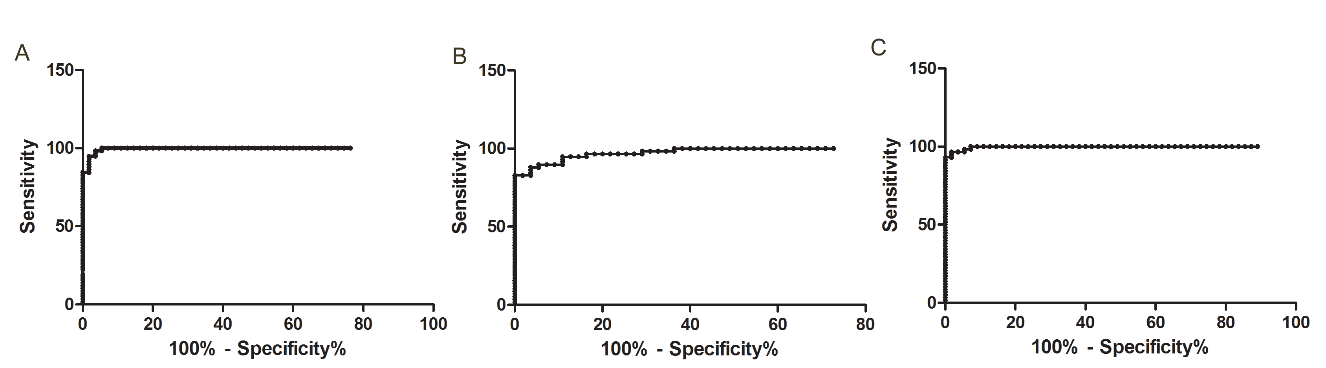


Figure S2 – ROC curves obtained from *r*Tc_11623.20 (A), *r*Tc_N_10421.310 (B) and a pool of these two recombinant proteins (C) as antigens with sera from Chagasic and non-chagasic human patients. These results were used to determinate the cutoff value in order to maximize sensitivity and specificity.
