# Supplementary material for: Genome-Wide Screening and Identification of New Trypanosoma cruzi Antigens with Potential Application for Chronic Chagas Disease Diagnosis
Source: PLoS One. 2014 Sep 16;9(9):e106304. doi: 10.1371/journal.pone.0106304 (PMC4165580; doi:10.1371/journal.pone.0106304)
Supplement: Table S2 — Primers used to amplify the entire coding region of the Tc00.1047053511623.20 gene and the 5′ end of the Tc00.1047053510421.310 gene. (DOCX) [file pone.0106304.s004.docx]

| **Recombinant protein code** | **Protein ID**  **(Tritryp DB)** | **Annotation**  **(Tritryp DB)** | **Amplicon (bp)** | **Primer sequence** | **Primer TM(^o^C)** | **Restriction site** |
| --- | --- | --- | --- | --- | --- | --- |
| ***r*Tc_11623.20** | Tc00.1047053511623.20 | Hypothetical protein conserved | 1215 | 5'-GCTAGCATGCTTGCTCAGGGTG-3' | 56,3 | *Nhe*I |
|  |  |  |  | 5'-AAGCTTCTATCGATGTTGAATTTGAAG-3' | 54 | *Hind*III |
| ***r*Tc_N_10421.310** | Tc00.1047053510421.310  (N-terminal portion) | Hypothetical protein conserved | 1704 | 5'-GCTAGCATGTACACATCACTGAACAG-3' | 51,2 | *Nhe*I |
|  |  |  |  | 5'-GAGCTCTCACAGCCGACGAATTTCAG'-3' | 58,8 | *Sac*I |

**Table S2 - Primers used to amplify the entire coding region of the Tc00.1047053511623.20 gene and the 5’ end of the Tc00.1047053510421.310 gene**
