# Supplementary material for: Genome-Wide Screening and Identification of New Trypanosoma cruzi Antigens with Potential Application for Chronic Chagas Disease Diagnosis
Source: PLoS One. 2014 Sep 16;9(9):e106304. doi: 10.1371/journal.pone.0106304 (PMC4165580; doi:10.1371/journal.pone.0106304)

**Table S3 – Reactivity of the top ten peptides in the immunoblotting assays with the sera from mice chronically infected with distinct *T.cruzi* strains.**


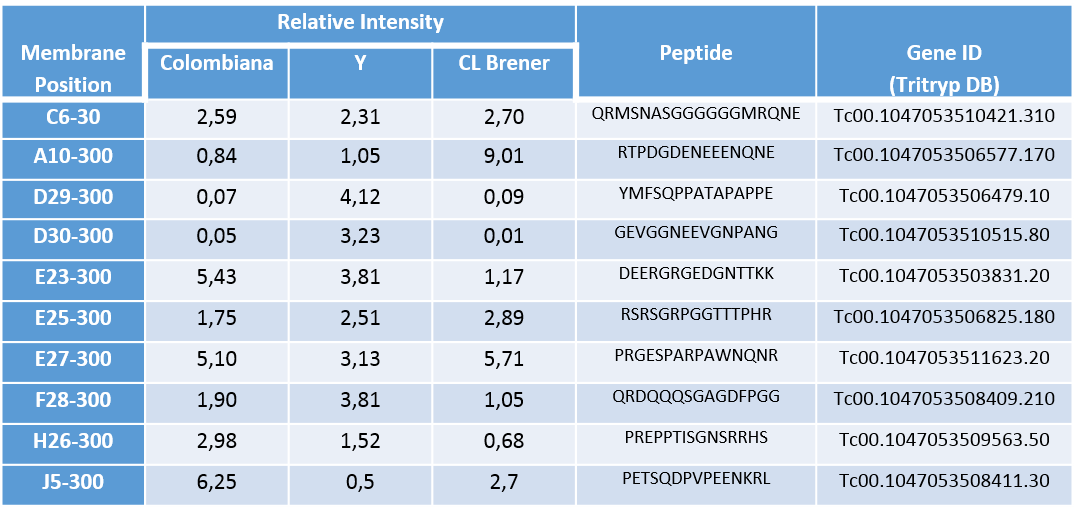

Supplement: Table S3 — Reactivity of the top ten peptides in the immunoblotting assays with the sera from mice chronically infected with distinct T. cruzi strains. (DOCX) [file pone.0106304.s005.docx]
