# Supplementary material for: Genome-Wide Screening and Identification of New Trypanosoma cruzi Antigens with Potential Application for Chronic Chagas Disease Diagnosis
Source: PLoS One. 2014 Sep 16;9(9):e106304. doi: 10.1371/journal.pone.0106304 (PMC4165580; doi:10.1371/journal.pone.0106304)
Supplement: Table S4 — Measure of diagnostic performance for rTc_11623.20, rTc_N_10421.310 and pooled antigens. (DOCX) [file pone.0106304.s006.docx]

**Table S4:** **Measure of diagnostic performance for *r*Tc_11623.20, *r*Tc_N_10421.310 and pooled antigens.**

**
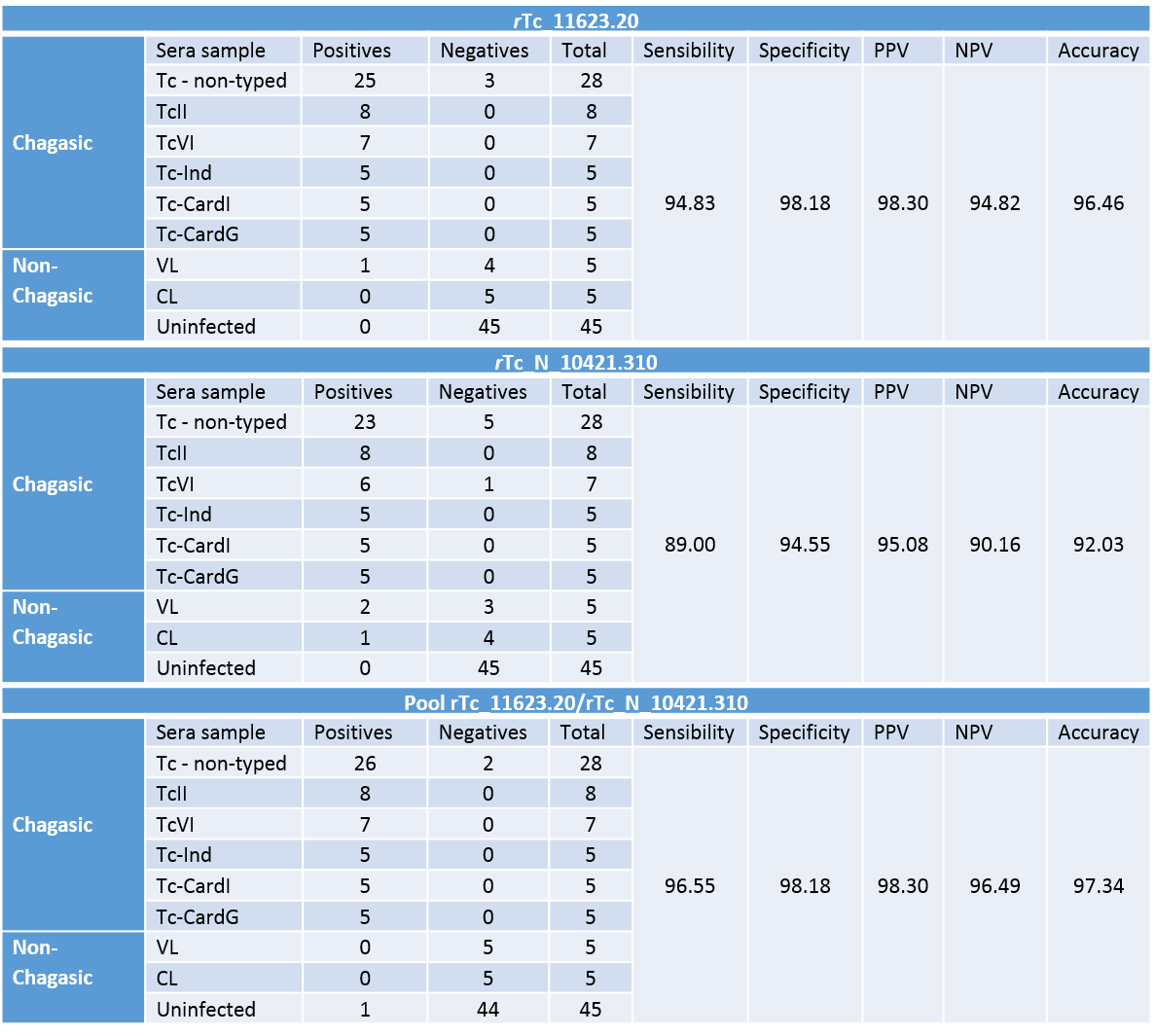
**

Values of sensitivity, specificity, positive predictive value (PPV), negative predictive value (NPV) and accuracy for each recombinant protein and pooled antigens are shown. Each row corresponds to a different group of sera assayed in the experiments. Tc-non typed, sera from chronic chagasic patients; TcII, sera from patients infected with *T. cruzi* TcII DTU; TcVI, sera from patients infected with *T. cruzi* TcVI DTU, Tc-Ind, sera from chagasic patients in the indeterminate form of Chagas disease; Tc-CardI, sera from chagasic patients in the initial cardiac stage of Chagas disease; Tc-CardG, sera from chagasic patients in the severe cardiac stage of Chagas disease; VL, sera from patients with visceral leishmaniasis; CL, sera from patients with cutaneous leishmaniasis.
